# 3D Printed Punctal Plugs for Controlled Ocular Drug Delivery

**DOI:** 10.3390/pharmaceutics13091421

**Published:** 2021-09-08

**Authors:** Xiaoyan Xu, Sahar Awwad, Luis Diaz-Gomez, Carmen Alvarez-Lorenzo, Steve Brocchini, Simon Gaisford, Alvaro Goyanes, Abdul W. Basit

**Affiliations:** 1 Department of Pharmaceutics, UCL School of Pharmacy, University College London, 29-39 Brunswick Square, London WC1N 1AX, UK; xiaoyan.xu.13@ucl.ac.uk (X.X.); s.awwad@ucl.ac.uk (S.A.); s.brocchini@ucl.ac.uk (S.B.); s.gaisford@ucl.ac.uk (S.G.); 2 Departamento de Farmacología, Farmacia y Tecnología Farmacéutica, I+D Farma (GI-1645), Facultad de Farmacia and Health Research Institute of Santiago de Compostela (IDIS), Universidade de Santiago de Compostela, 15782 Santiago de Compostela, Spain; luis.diaz.gomez@usc.es (L.D.-G.); carmen.alvarez.lorenzo@usc.es (C.A.-L.); 3 FabRx Ltd., Henwood House, Henwood, Ashford, Kent TN24 8DH, UK

**Keywords:** additive manufacturing, vat photopolymerization, digital light processing, printing pharmaceuticals and devices, punctal plugs, ocular drug delivery, personalized medicines, dry eye

## Abstract

Dry eye disease is a common ocular disorder that is characterised by tear deficiency or excessive tear evaporation. Current treatment involves the use of eye drops; however, therapeutic efficacy is limited because of poor ocular bioavailability of topically applied formulations. In this study, digital light processing (DLP) 3D printing was employed to develop dexamethasone-loaded punctal plugs. Punctal plugs with different drug loadings were fabricated using polyethylene glycol diacrylate (PEGDA) and polyethylene glycol 400 (PEG 400) to create a semi-interpenetrating network (semi-IPN). Drug-loaded punctal plugs were characterised in terms of physical characteristics (XRD and DSC), potential drug-photopolymer interactions (FTIR), drug release profile, and cytocompatibility. In vitro release kinetics of the punctal plugs were evaluated using an in-house flow rig model that mimics the subconjunctival space. The results showed sustained release of dexamethasone for up to 7 days from punctal plugs made with 20% *w*/*w* PEG 400 and 80% *w*/*w* PEGDA, while punctal plugs made with 100% PEGDA exhibited prolonged releases for more than 21 days. Herein, our study demonstrates that DLP 3D printing represents a potential manufacturing platform for fabricating personalised drug-loaded punctal plugs with extended release characteristics for ocular administration.

## 1. Introduction

Vision is considered one of the most important senses [1]. Dry eye is a common chronic disorder that affects millions of people worldwide and represents a growing public health concern [2,3]. It occurs due to deficient tear production and/or increased evaporation of the tear film, and can lead to corneal inflammation and conjunctiva if left untreated. Ocular drug delivery has always been a challenging task because of the static and dynamic barriers that provide protection against external agents reaching the eye, such as pathogens and therapeutic molecules [4,5]. Topical administration (e.g., eye drops) is the preferred route for the delivery of therapeutic agents to the anterior segment of the eye because it minimises systemic side effects, is non-invasive, and is easily accessible. However, ocular bioavailability from topically applied formulations is usually poor (<5%), a result of numerous factors, including short drug residence time, blinking, high tear turnover rate and naso-lacrimal drainage [4].

Novel formulation approaches to improve ocular bioavailability include extending the drug residence time or promoting corneal penetration with the use of liposomes, nanoparticles, penetration enhancers, mucoadhesive polymers and/or in situ gelling components, although maintaining stability could be challenging [6,7,8]. Physical force-based methods, such as iontophoresis and sonophoresis, represent promising strategies to enhance penetration efficiency by temporarily disrupting the barrier structures in a minimally or non-invasive fashion [9]. Concerns about temporary tissue damage from these strategies are present [7,10]. A variety of state-of-the-art drug-eluting systems have been developed for effective and extended delivery of ocular therapeutics release, including microneedles [11,12], drug-eluting contact lenses [7,13], and nanowafers [14,15].

Use of punctal plugs is a common, non-invasive treatment strategy to mitigate dry eye syndromes [16,17]. They work by blocking the canaliculi, which connects the eyes to the nose, preventing tear drainage [6,18,19]. Punctal occlusion by means of plugs has been reported to improve tear film stability, tear osmolarity, and functional visual acuity of dry eye patients [20]. In general, punctal plugs can be classified based on their location: in the puncta or in the canaliculus (Figure S1). Punctal plugs that are inserted at the opening of the puncta are visible and easily removable after treatment. In contrast, canalicular plugs are invisible, as they are placed in the horizontal or vertical canaliculus, which makes removal difficult. Temporary punctal plugs are normally made from collagen and last for up to 14 days, whereas permanent plugs are usually made of polydioxanone and polycaprolactone (PCL) and can last from 2–6 months [18].

Three-dimensional (3D) printing is a versatile additive manufacturing technology that enables rapid prototyping of bespoke objects created using computer-aided design (CAD) software or imaging techniques [21] across many fields including the aerospace [22] and food industries [23,24], robotics [25], and biomedicine [26,27]. In recent years, 3D printing has been forecast to become a revolutionary technology within the pharmaceutical sector [28] because of its capability of fabricating small batches of personalised medicines with unconventional designs [29,30,31,32,33], drug combinations [34,35,36,37,38], and tailored release characteristics [39,40,41,42] as well as patient-specific medical devices [43,44,45,46,47]. Among different 3D printing technologies, vat photopolymerisation-based 3D printing (such as digital light processing (DLP), stereolithography (SLA), and continuous liquid interface production (CLIP)) is a type of 3D printing technique that creates solid objects via photopolymerisation of a vat of liquid resin upon light irradiation in a layer-by-layer fashion [48,49]. This type of technology offers remarkable feature resolution and a smooth surface finish that other 3D printing technologies cannot achieve. In addition, it operates at room temperature; hence, it avoids the risk of thermal degradation of drugs [50]. Thus far, vat photopolymerisation 3D printing techniques have been explored to prepare pharmaceutical products for various drug delivery purposes, including oral dosage forms [51,52,53], nose patches [45], microneedles [54,55,56], intravaginal rings [57], dental [58], bladder devices [59] and hearing aids [60].

The aim of this work was to employ DLP 3D printing to fabricate drug-loaded punctal plugs for controlled drug delivery. DLP 3D printing can be particularly advantageous, since it is possible to prepare various sizes, shapes, and doses of the punctal plugs in a single step. Here, dexamethasone, a corticosteroid, was incorporated because of its anti-inflammatory properties and wide applications in corneal disease treatment, including dry eye symptoms [61,62]. Different formulations of drug-loaded punctal plugs were prepared in polyethylene glycol diacrylate (PEGDA) hydrogels via direct DLP 3D printing, followed by physical characterisations and in vitro drug release studies using an in-house developed in vitro ocular flow model of the subconjunctival space.

## 2. Materials and Methods

### 2.1. Materials

Dexamethasone (MW 392.46 g/mol, Pharmaceutical Secondary Standard), phenylbis (2,4,6-trimethylbenzoyl) phosphine oxide (Irgacure 819, MW 418.46 g/mol), β-carotene (MW 536.87 g/mol, ≥93% UV), polyethylene glycol P400 (PEG 400), and polyethylene glycol diacrylate (PEGDA, Mn 575), Dulbecco’s phosphate buffered saline (PBS), trifluoroacetic acid (TFA, ≥99.0%, HPLC grade), and acetonitrile (ACN, ≥99.9%, HPLC grade) were obtained from Sigma-Aldrich (Dorset, UK). All materials were used as received.

### 2.2. Methods

#### 2.2.1. Design of the Punctal Plug

**Figure 1:**
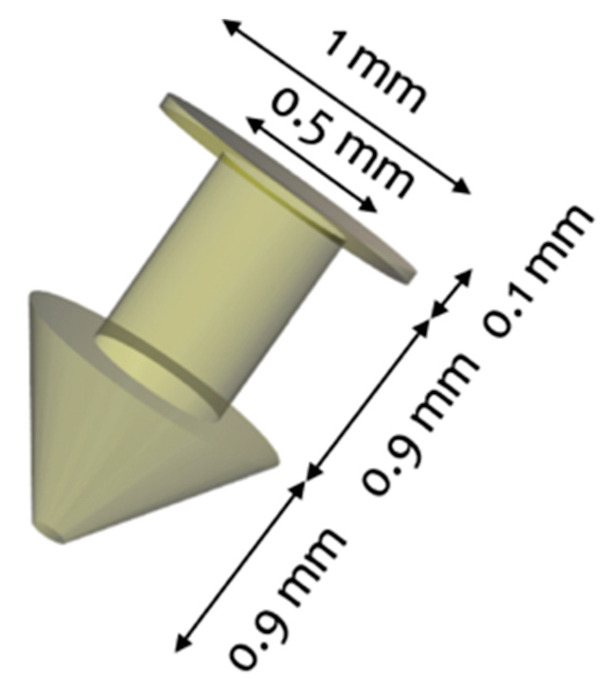
3D design of the punctal plug.

The punctal plug was designed to be inserted in the punctum of the eye with a configuration similar to those of commercially available punctal plugs, which range from 1.0–2.0 mm in length and from 0.2–1.0 mm in diameter. The dimension of the punctal plug was 1.9 mm in length and 1.0 mm in diameter with a cylindrical core diameter of 0.5 mm (Figure 1).

#### 2.2.2. Preparation of Photopolymer Solutions

Photopolymer solutions were prepared with 2.0% (*w*/*w*) photoinitiator (Irgacure 819) and 1.0% (*w*/*w*) photoabsorber (β-carotene) to a total mass of 2.0 g. Dexamethasone, PEGDA, and PEG 400 were added to each photopolymer solution according to the compositions shown in Table 1. In formulations D10 and D20, only PEGDA was used as polymeric component, while in the formulations D10PEG and D20PEG, PEG 400 was added to form the semi-interpenetrating network (semi-IPN) and facilitate drug release (PEGDA:PEG 400 4:1 *w*/*w* ratio). The photopolymer solutions were kept in amber containers and continuously stirred overnight at room temperature (~25 °C). The solution was then added into the resin tank of the DLP 3D printing.

#### 2.2.3. Printing Process

**Figure 2:**
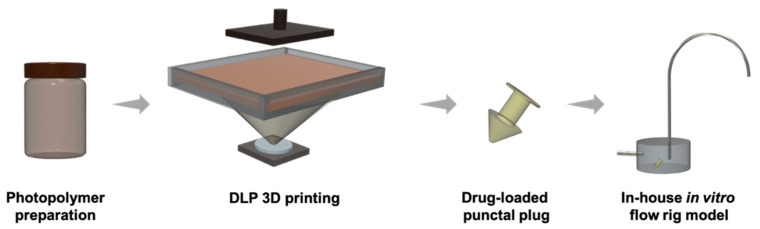
Schematic representation of the DLP 3D printing process and in-house flow rig model for in vitro dissolution studies.

All the punctal plugs were printed with a commercial DLP 3D printer (Titan2 HR, Kudo3D Inc., Dublin, CA, USA) equipped with an HD DLP projector, which has a visible light source (400–700 nm) (Figure 2). The printer XY resolution was set to 23 μm. The punctal plugs were designed with 123D Design (Autodesk Inc., Mill Valley, CA, USA) and exported as a stereolithographic file (.stl) into the Kudo3D Print Job software for slicing into layers with a thickness of 25 μm. These files were subsequently sent for printing. All punctal plugs were printed with generated supports from the Kudo software at a 45° angle on the build platform. The printing time was 2 s per layer for the D10 and D10PEG punctal plugs and 5 s per layer for the D20 and D20PEG punctal plugs (10 s for the first layer). After printing, the plugs were rinsed for 1 min in isopropyl alcohol (IPA) in the sonicator, followed by a post-cure process at 405 nm for 30 min in a Form Cure (Formlabs Inc., Somerville, MA, USA). The total printing time was 20 min.

#### 2.2.4. UV-Visible Spectrophotometry

Photoinitiator (Irgacure 819) and photoabsorber (β-carotene) solutions were prepared at concentrations of 0.025% (*w*/*v*) and 0.0025% (*w*/*v*) in ethanol, respectively. The UV-visible spectra were analysed on a Cary 100 UV–vis spectrophotometer (Agilent Technologies, Cheshire, UK) between 200–800 nm at a scan rate of 600 nm/min.

#### 2.2.5. X-ray Powder Diffraction (XRPD)

Discs (23.0 mm diameter × 1.0 mm height) were specifically prepared for XRPD analysis for different formulations (D10, D20, D10PEG, D20PEG) using the same printing conditions as the drug-loaded punctal plugs. Powdered samples of pure dexamethasone were analysed by XRPD. XRPD patterns were obtained with a Rigaku MiniFlex 600 (Rigaku, Wilmington, MA, USA) equipped with a Cu Kα X199 ray source (λ = 1.5418 Å). The intensity and voltage applied were 15 mA and 40 kV. Samples were scanned between 2θ = 3–60° with a stepwise size of 0.02° at a speed of 5°/min.

#### 2.2.6. Differential Scanning Calorimetry (DSC)

DSC measurements were performed with a Q2000 DSC (TA instruments, Waters, LLC, New Castle, DE, USA) at a heating rate of 10 °C/min from 0 to 300 °C to characterise dexamethasone powder and different punctal plugs (D10, D20, D10PEG, D20PEG). Nitrogen was used as a purge gas with a flow rate of 50.0 mL/min for all the experiments. Data were collected with TA Advantage software for Q series (version 2.8.394, TA instruments, Waters LLC, New Castle, DE, USA) and analysed using TA Instruments Universal Analysis 2000. TA aluminium pans and pin-holed hermetic lids (T_zero_) were used with an average sample size of 3.0–5.0 mg.

#### 2.2.7. Device Morphology

Images of the DLP 3D printed punctal plugs (D10, D20, D10PEG, D20PEG) were captured with a Leica Galen III optical microscope (Leica, Wetzlar, Germany) and photographs were taken with an iPhone (Apple, Los Altos, CA, USA) through the eyepiece. The length of the punctal plugs were measured using ImageJ (NIH, Bethesda, MD, USA) (*n* = 3).

#### 2.2.8. Scanning Electron Microscopy (SEM)

DLP 3D printed punctal plugs (D10, D20, D10PEG, D20PEG) were attached to a self-adhesive carbon disc mounted on a 25.0 mm aluminium stub, which was sputter coated with 25.0 nm of gold. The images were captured with an FEI Quanta 200 FEG SEM (FEI, Hillsboro, OR, USA) at 5.0 kV accelerating voltage using secondary electron detection to obtain the images.

#### 2.2.9. Fourier-Transform Infrared Spectroscopy (FTIR)

The infrared spectra of formulations (D10, D20, D10PEG, D20PEG) before and after DLP 3D printing were collected using a Spectrum 100 FTIR spectrometer (PerkinElmer, Waltham, MA, USA). All samples were scanned over a range of 4000–650 cm^−1^ at a resolution of 1 cm^−1^ for 6 scans. The spectra of pure dexamethasone drug powder and PEGDA were collected as the references.

#### 2.2.10. In Vitro Drug Release from the Devices

Drug release rates from the punctal plugs were measured using an in-house in vitro dynamic flow cell model (Figure 2) [63]. A sample chamber (8.8 mm diameter × 3.27 mm height) with a capacity of 200.0 µL was employed for the study, and the punctal plug was placed in the chamber. In order to simulate the condition in the front of the eye, fresh PBS (pH 7.4, with 0.05% sodium azide, 37 °C) was used as the medium, which was continuously supplied by a peristaltic pump (Michael Smith Engineers Ltd., Woking, UK) at a flow-rate of 1.6–2.0 µL/min via the inlet port into the chamber. Samples were collected from the outlet port for a period of 21 days. Dexamethasone release was determined using high performance liquid chromatography (HPLC) at 240 nm.

#### 2.2.11. Determination of Drug Content

The concentration of dexamethasone in the DLP 3D printed punctal plugs was determined by cutting the plugs into small pieces. They were stirred in 5.0 mL ethanol at room temperature overnight. The solutions were filtered through a 0.45 μm filter (Millipore Ltd., Cork, Ireland), and drug concentration was determined with HPLC (Hewlett Packard 1260 Series HPLC system, Agilent Technologies, Cheadle, UK). An Eclipse plus C18 column, 100 × 4.6 mm (Zorbax, Agilent technologies, Cheshire, UK) was employed with a flow-rate of 1.0 mL/min and a mobile phase gradient consisting of ACN and 0.1% *v*/*v* TFA water. The gradient was: 20% ACN increased to 70% in 10 min, then decreased to 20% in 1 min and held for 4 min prior to the next injection. The sample injection volume was 100 μL operating at 30 °C at a detection wavelength of 240 nm.

#### 2.2.12. Cytocompatibility Studies

BalB/3T3 cells (CCL-163; ATCC, Manassas, VA, USA) were used to evaluate the cytotoxicity of different 3D printed discs (6 mm diameter × 1 mm height) prepared with the drug-loaded formulations (D10, D20, D10PEG, D20PEG) and the control formulations (C-PEGDA and C-PEGDA:PEG). Cells were cultured at 37 °C in 75 cm^2^ flasks using complete culture medium (DMEM supplemented with 10% *v*/*v* fetal bovine serum (FBS) and 1% *v*/*v* penicillin/streptomycin/fungizone (PSF)) until 80% confluence was reached. Cells were then trypsinized using TripLE solution (Sigma-Aldrich, St. Louis, MO, USA) and seeded in wells of 24 well plates at a concentration of 3 × 10^4^ cells per well. After 24 h, the discs (*n* = 5) were placed individually in wells, and plates were returned for incubation at 37 °C for 24 or 48 h. At each timepoint, medium was discarded and cells were washed with PBS. Freshly prepared CCK8 working solution (400 μL) consisting of culture medium (360 μL) and CCK-8 reagent (40 μL) (Dojindo; Tokyo, Japan) was added to each well and incubated for 2 h at 37 °C. Absorbance was recorded at 450 nm using a microplate reader (Model 8; BioRad, Hercules, CA, USA).

## 3. Results and Discussion

### 3.1. DLP 3D Printing

**Figure 3:**
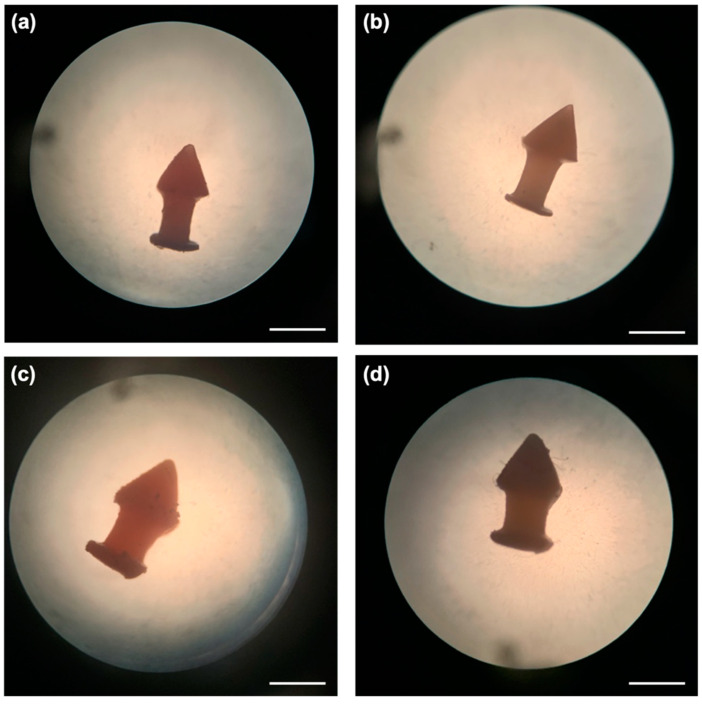
Light microscope images of the DLP 3D printed (**a**) D10, (**b**) D10PEG, (**c**) D20, and (**d**) D20PEG punctal plugs. The scale bar is equivalent to 1 mm.

Different dexamethasone-loaded punctal plugs were successfully fabricated via DLP 3D printing with good resolution and reproducibility. The punctal plug was designed to possess a tapered shaft, which exerts horizontal force to keep it in place for easy insertion and removal [18]. The total printing time was only 20 min and more than 20 punctal plugs could be fabricated in one print, highlighting the capability of 3D printing in the preparation of small-batch personalised drug delivery devices. The punctal plugs showed an orange colour due to the addition of β-carotene (Figure 3). All the devices were fabricated with uniform weights and dimensions (Table 2).

**Figure 4:**
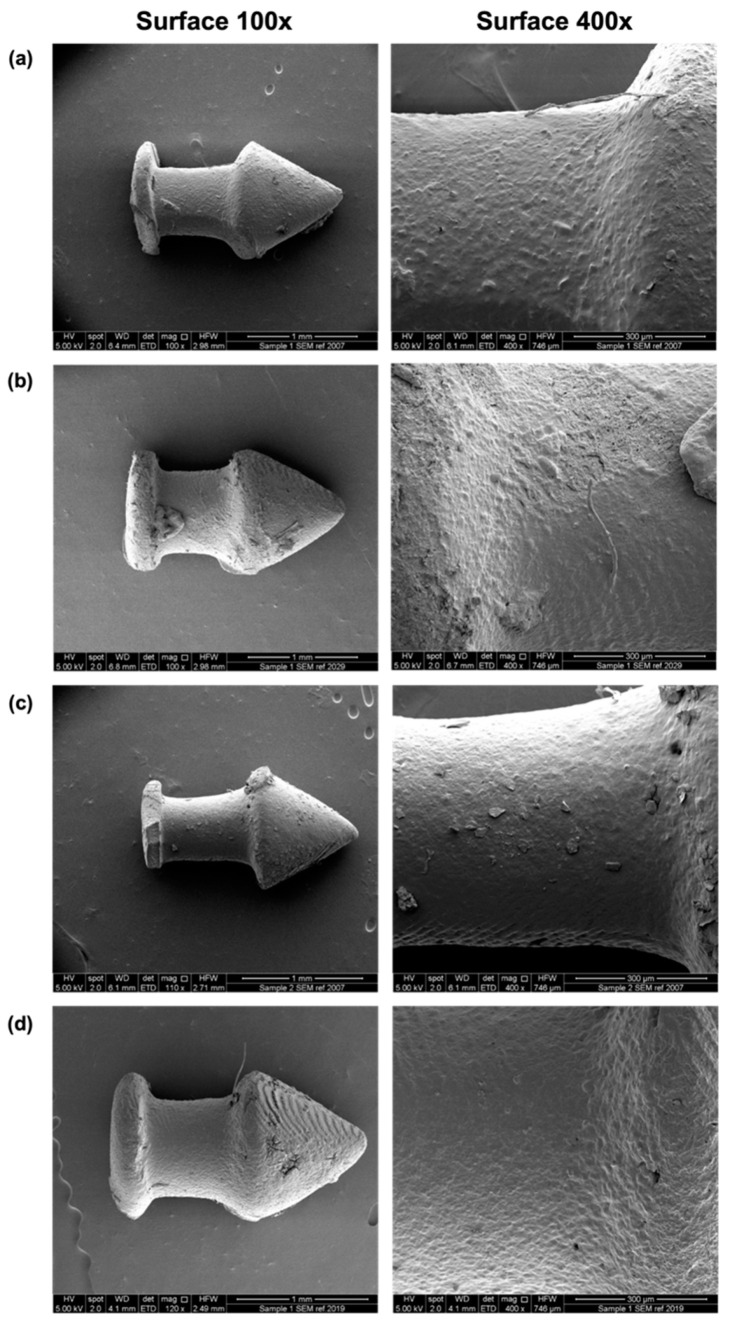
SEM images of the DLP 3D printed (**a**) D10, (**b**) D20, (**c**) D10PEG and (**d**) D20PEG punctal plugs. The scale bar is equivalent to 1 mm (surface 100×) and 300 µm (surface 400×).

SEM images showed the surface morphologies of the different punctal plugs (Figure 4). A minor increase in roughness was seen with an increase in drug loading from 10 to 20%. Supports were used due to the overhanging structure of the punctal plug. Although the removal of supports after printing was easily achieved using tweezers, minor damage was visualised on the surface (Figure 4a,c).

The DLP 3D printer used in this study was equipped with a visible-light source (400–700 nm), which offers numerous benefits over UV light, such as being more energy efficient with a reduced risk of eye damage and improved biocompatibility and functional group tolerance [64,65,66]. Here, PEGDA was selected because it is one of the most commonly used photocurable materials for biomedical applications [67,68,69]. In the photopolymer solutions, β-carotene was incorporated as a photo absorber due to its absorbance in the visible light range (400–500 nm). Unlike commonly used photo absorbers, such as Sudan I, which have been reported to be genotoxic and carcinogenic [70], β-carotene is a natural pigment found in plants and fruits and acts as an antioxidant, and so it represents a suitable choice as a biocompatible photo absorber [71,72].

### 3.2. Physical Characterisation of the 3D Printed Formulations

**Figure 5:**
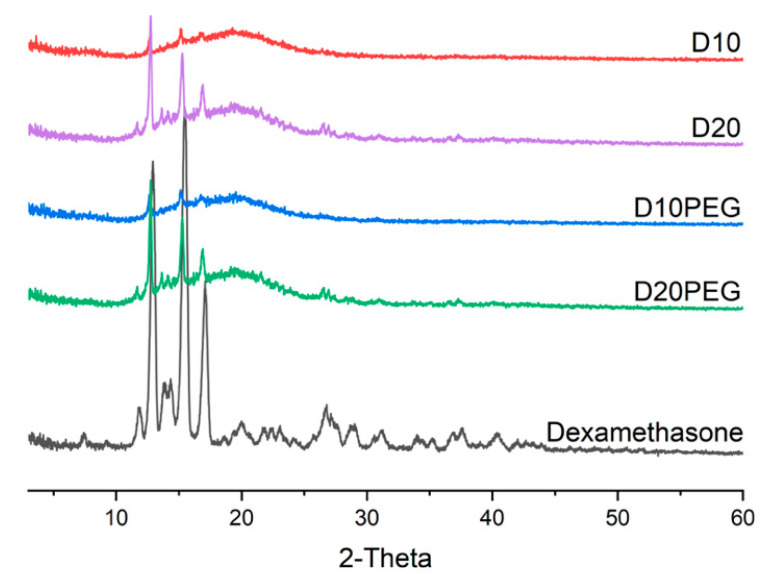
X-ray powder diffraction patterns of dexamethasone and DLP 3D printed formulations.

Dexamethasone and DLP 3D printed drug-loaded discs (23.0 mm diameter × 1.0 mm height) for different formulations (D10, D20, D10PEG, D20PEG) were analysed by XRPD to evaluate the physical state of the drug and the degree of its incorporation in the polymer matrices. The diffraction patterns (Figure 5) show characteristic peaks of dexamethasone at 12.92° 2θ, 15.50° 2θ, and 17.08° 2θ in the printed formulations, suggesting part of the drug remained in a crystalline state. Peaks were more intense in the D20 and D20PEG 3D printed formulations when the drug content was increased from 10 to 20% (*w*/*w*), indicating a greater proportion of drug was crystalline.

**Figure 6:**
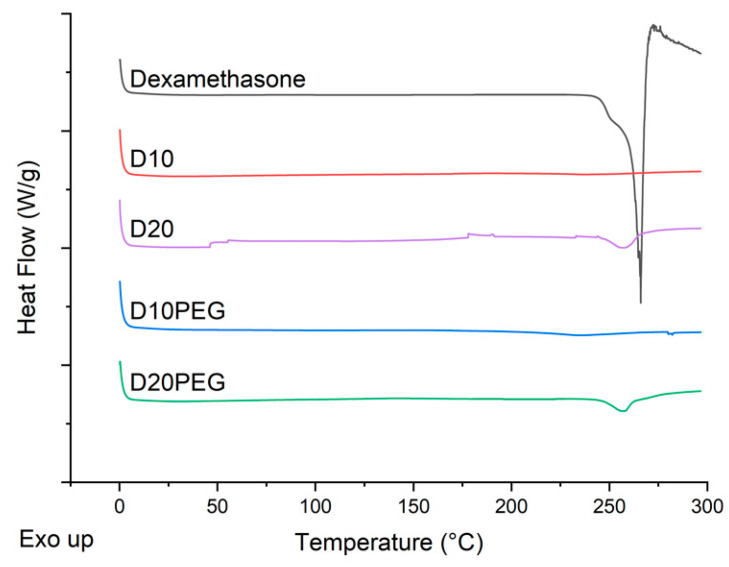
DSC scans of dexamethasone and DLP 3D printed formulations.

DSC thermograms were also obtained for the dexamethasone powder and different punctal plugs (D10, D20, D10PEG, D20PEG), showing the melting peak of dexamethasone at 266 °C (Figure 6), in agreement with literature [73]. No evidence of melting was observed in the D10 or D10PEG 3D printed formulations. Both D20 and D20PEG showed a small endothermic peak at 258 °C, consistent with there being a small fraction of drug in the crystalline state, as also noted in the XRPD data.

**Figure 7:**
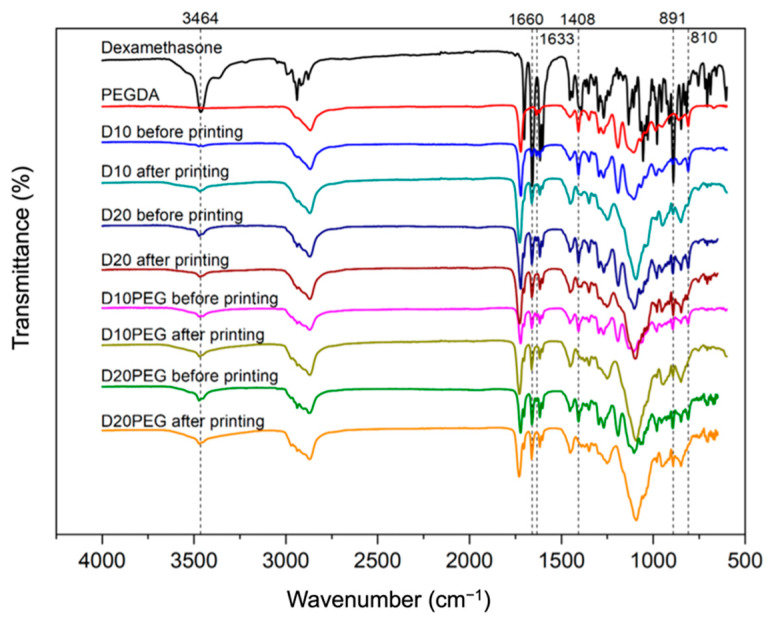
FTIR spectra of dexamethasone, PEGDA, and different formulations before and after DLP 3D printing.

FTIR was performed to investigate potential interactions between dexamethasone and the photopolymer before and after DLP 3D printing. Previously, an unexpected drug-photopolymer reaction has been reported in oral dosage forms prepared using SLA 3D printing [36]. Therefore, it is important to ensure the drug-photopolymer compatibility of the fabricated drug-loaded punctal plugs in order to maintain therapeutic efficacy. Characteristic peaks of dexamethasone (Figure 7) were recorded at 3464 cm^−1^ (O–H stretching), 1660 cm^−1^ (C=O stretching), and 891 cm^−1^ (axial deformation of C–F group) [74]. These absorption bands were clearly observed in the spectra of all the formulations before and after 3D printing, indicating the presence of the drug, and no interactions were seen between dexamethasone and the photopolymers. For comparison, the spectrum of PEGDA is also included, showing its distinct peaks at 1722 cm^−1^ (C=O stretching), 1633 cm^−1^ (C=C stretching), 1408 cm^−1^ and 810 cm^−1^ (CH_2_=CH) [75,76]. After 3D printing, these peaks at 1633 cm^−1^, 1408 cm^−1^ and 810 cm^−1^ disappeared because of the conversion of C=C bonds to C–C bonds via photocrosslinking [51,77,78].

### 3.3. Drug Release Studies

**Figure 8:**
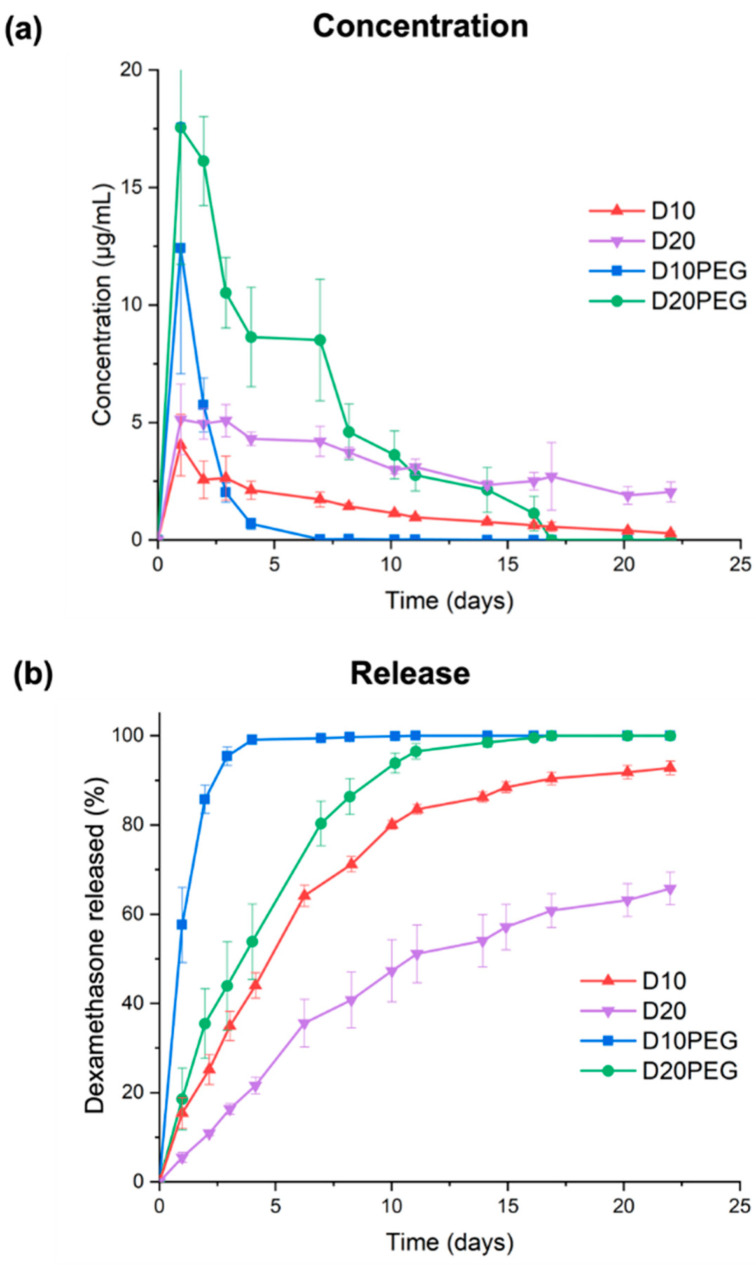
(**a**) Concentration and (**b**) cumulative release profile of dexamethasone of the DLP 3D printed punctal plugs in a rig model mimicking the subconjunctival space (~2.0 µL/min, 37 °C). Data are shown as mean ± SD.

Drug loadings of the different DLP 3D printed punctal plugs were determined using HPLC. The loading of dexamethasone in various DLP 3D printed punctal plugs was consistent with that in the photopolymer solution (Table 3). Dissolution studies were conducted using an in-house in vitro model that mimics the subconjunctival space of the eye [63]. Fresh PBS (pH 7.4) was constantly pumped into the inlet port at a rate of 1.6–2.0 µL/min to mimic the aqueous turnover rate of the human eye. A burst release of dexamethasone with a C_max_ value of 12.4 ± 5.3 µg/mL (57.6 ± 8.4% of total drug released) was observed by day 1 from the D10PEG punctal plugs (Figure 8). Complete drug release was achieved in 4 days. On the other hand, the D20PEG punctal plugs exhibited a polyphasic release profile, with a burst concentration at 16.1–17.6 µg/mL in the first 2 days, followed by a constant rate of release at 8.5–10.5 µg/mL in the next 5 days (accounting for 80.4 ± 5.0% of release in 1 week). The concentration was maintained at approximately 3.0 µg/mL per day in the remaining period. Although the reduced PEGDA concentration in the D20PEG punctal plugs was expected to lower the polymer density in the crosslinked network (and increase the drug release rate), the observed slower dissolution rate from D20PEG was mainly attributed to the poor aqueous solubility of dexamethasone (0.1 mg/mL) [79].

Conversely, prolonged release profiles were observed for the D10 and D20 punctal plugs. Despite the C_max_ reaching 4.0 ± 1.3 µg/mL in the first day, D10 displayed a release rate ranging from 2.6–1.7 µg/mL in the first 7 days, reaching 64.1 ± 2.4% of dexamethasone release. D20 demonstrated a continuous and monophasic release profile over 21 days, with a close-to-constant release rate between 2.1–5.1 µg/mL, where only 51.1 ± 6.5% of total release was achieved within 11 days. As expected, the incorporation of PEG 400 as a hydrophilic diluent in D10PEG and D20PEG lowered the polymer density in the matrix, facilitating the drug release rates [77,80]. Recently, dexamethasone-loaded nanowafers [15] were proposed for treating dry eye disease; however, drug release only continued for 24 h.

**Figure 9:**
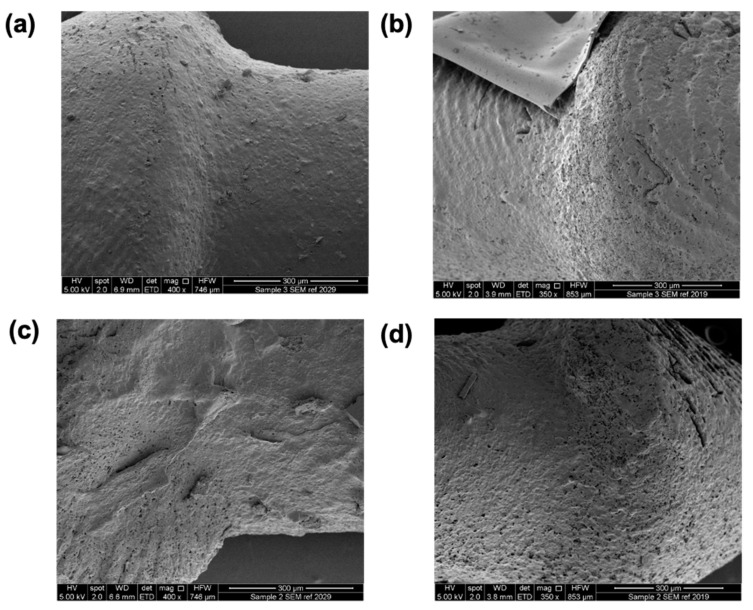
SEM images of DLP 3D printed (**a**) D10, (**b**) D10PEG, (**c**) D20, and (**d**) D20PEG punctal plugs after dissolution.

After the 21-day release study, the punctal plugs were collected, dried and imaged with SEM (Figure 9). Compared with the SEM images before dissolution (Figure 4), porous surfaces can be observed in all the plugs, contributing to the release of dexamethasone from the devices. The punctal plugs with higher drug loading (D20 and D20PEG) showed an increased number of pores than the D10 and D10PEG plugs.

### 3.4. Cytocompatibility Studies

**Figure 10:**
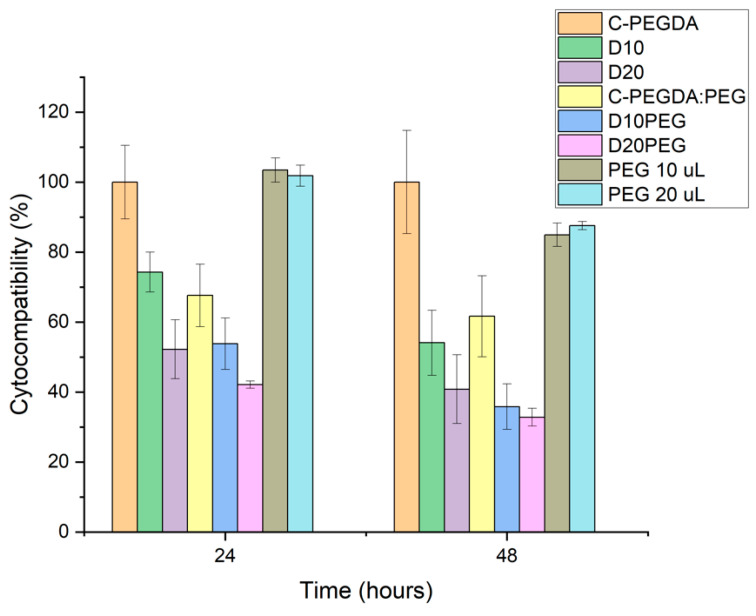
Viability of Balb/3T3 cells after 24 and 48 h in contact with C-PEGDA, D10, D20, C-PEGDA:PEG, D10PEG, D20PEG DLP 3D printed samples, and PEG 400 (10 µL and 20 µL).

The cytocompatibility of the DLP 3D punctal plugs was evaluated by direct contact using a BALB/3T3 fibroblast cell line, according to the international standard ISO 10993-5 [81] (Figure 10). Blank plugs prepared with 100% PEGDA (C-PEGDA) and 80% PEGDA:20% PEG400 (C-PEGDA:PEG) were used as controls. Complete (100%) cell viability was observed for C-PEGDA (control) after 24 h incubation, similar to previously evaluated PEGDA constructs [82]. Conversely, the cytocompatibility of samples containing dexamethasone was significantly decreased (*p* < 0.05) as compared to the C-PEGDA control, i.e., 74.3 ± 5.7% for D10 and 52.2 ± 8.4% for D20 in 24 h, and 54.1 ± 9.3% for D10 and 40.8 ± 9.8% for D20 in 48 h. On the other hand, the viability of the fibroblasts significantly decreased to 67.6 ± 8.9% and 61.6 ± 11.6% after incubation in 20% PEG 400 in the C-PEGDA:PEG controls in 24 and 48 h (*p* < 0.05). A decrease of approximately 50% in cell viability was shown in D10PEG and D20PEG plugs after 24 h, indicating the composition-dependent cytotoxicity of the formulations. From the drug release results (Figure 8), all the drug-loaded punctal plugs were expected to release certain amounts of dexamethasone in the cell culture medium, which was reflected with the low cell proliferation values—the cytotoxic effect of free dexamethasone (1 mM) has been previously reported in literature [83,84]. Although the molecular weight and concentration of PEG 400 used in this study should not compromise cell viability, its incorporation in the composition could have lowered the crosslinking efficiency and, therefore, the release of unreacted monomers possessing potential cytotoxic effects [85]. The in vitro cellular cytotoxicity results suggested that punctal plugs prepared with PEG 400 in the photopolymer may require extra post-washing and post-curing steps to improve the conversion of monomers, and hence the biocompatibility.

Punctal plugs represent the most common non-medical treatment in moderate and severe dry eye syndrome. Previously, a few studies reported the combination use of eye drops and punctal plugs could provide an additive effect, indicating the evident advantages of developing drug-loaded punctal plugs [6,86]. In this study, we describe for the first time the possibility of using DLP 3D printing in the direct preparation of drug-loaded punctal plugs, offering vast opportunities in delivering ocular therapeutics effectively. The manufacturing process is one step and simple under mild conditions, which compares favourably with previously described drug-loaded punctal plugs [16,17]. Compared with traditional methods of preparing punctal plugs, such as moulding, DLP 3D printing offers high flexibility in personalising the size, shape, and material of the plug to suit different patients’ needs. Spontaneous extrusion has been previously reported by patients due to the use of non-optimal plug size [87]; therefore, the need to customise punctal plugs presents an opportunity. Numerous novel drug-eluting systems have been developed for delivering drugs to the front of the eye, for example, drug-laden contact lenses [13]. Nonetheless, the prolonged wear of hydrogel lens may reduce oxygen permeability, especially overnight, which could lead to corneal edema. Overall, this study highlights the potential of drug-releasing punctal plugs, which are not limited to dry eye disease, but could vastly benefit a range of ocular diseases, including open-angle glaucoma, ocular hypertension, and bacterial conjunctivitis [88,89].

## 4. Conclusions

Here, we report on a DLP 3D printing platform that enables the fabrication of drug-loaded punctal plugs with prolonged drug delivery to the front of the eye for patients with dry eye disease. All punctal plugs were successfully prepared with two loadings of dexamethasone (10% *w*/*w* and 20% *w*/*w*) using different compositions of PEGDA and PEG 400. The FTIR spectra confirmed the absence of drug-photopolymer interactions. In vitro drug release results showed that drug release from D10PEG and D20PEG plugs can be sustained for 1 week, while D10 and D20 plugs can prolong the release with 50% of dexamethasone released in 5 and 11 days, respectively. Drug-loaded punctal plugs were less cytocompatible than blank controls, possibly due to the release of dexamethasone and the inclusion of PEG 400. In summary, the possibility presented in this study in terms of fabricating personalised drug-loaded punctal plugs via DLP 3D printing allows us to extend opportunities by adapting other ocular therapeutics for drug delivery to the front eye.

## Figures and Tables

**Table 1:** Compositions (% *w*/*w*) of the drug-loaded photopolymer resins.

Formulations	Dexamethasone (%)	Irgacure 819 (%)	β-Carotene (%)	PEGDA (%)	PEG 400 (%)
D10	10.0	2.0	1.0	87.0	0.0
D20	20.0	2.0	1.0	77.0	0.0
D10PEG	10.0	2.0	1.0	69.6	17.4
D20PEG	20.0	2.0	1.0	61.6	15.4

**Table 2:** Weights and dimensions of different DLP 3D printed punctal plugs.

Formulations	Weight (mg ± SD)	Length (mm ± SD)
D10	0.86 ± 0.03	1.92 ± 0.03
D20	1.50 ± 0.03	2.02 ± 0.02
D10PEG	0.59 ± 0.05	1.81 ± 0.05
D20PEG	1.39 ± 0.03	2.04 ± 0.01

**Table 3:** Drug concentration in photopolymer solution and DLP 3D printed punctal plugs.

Formulations	Drug Loading (%, *w*/*w* ± SD)		Dose (µg)
	Photopolymer Solution	DLP 3D Printed Punctal Plugs	
D10	11.9 ± 0.3	9.8 ± 0.5	84.4
D20	20.2 ± 1.6	20.3 ± 2.1	305.6
D10PEG	10.6 ± 0.1	10.0 ± 0.6	59.0
D20PEG	19.4 ± 2.1	21.2 ± 1.4	294.4

## Supplementary Materials

The following are available online at https://www.mdpi.com/article/10.3390/pharmaceutics13091421/s1, Figure S1: Schematic illustration of punctal plug (a) in the punctum and (b) in the canaliculus of the eye.
