# Supplementary material for: 3D Printed Punctal Plugs for Controlled Ocular Drug Delivery
Source: Pharmaceutics. 2021 Sep 8;13(9):1421. doi: 10.3390/pharmaceutics13091421 (PMC8464872; doi:10.3390/pharmaceutics13091421)
Supplement: Supplementary file 1 [file pharmaceutics-13-01421-s001.zip › pharmaceutics-1360299-supplementary.pdf]

pharmaceutics

MDPI

Supplementary Materials: 3D Printed Punctal Plugs for Controlled Ocular Drug Delivery

Xiaoyan Xu, Sahar Awwad, Luis Diaz-Gomez, Carmen Alvarez-Lorenzo, Steve Brocchini, Simon Gaisford, Alvaro Goyanes and Abdul W. Basit

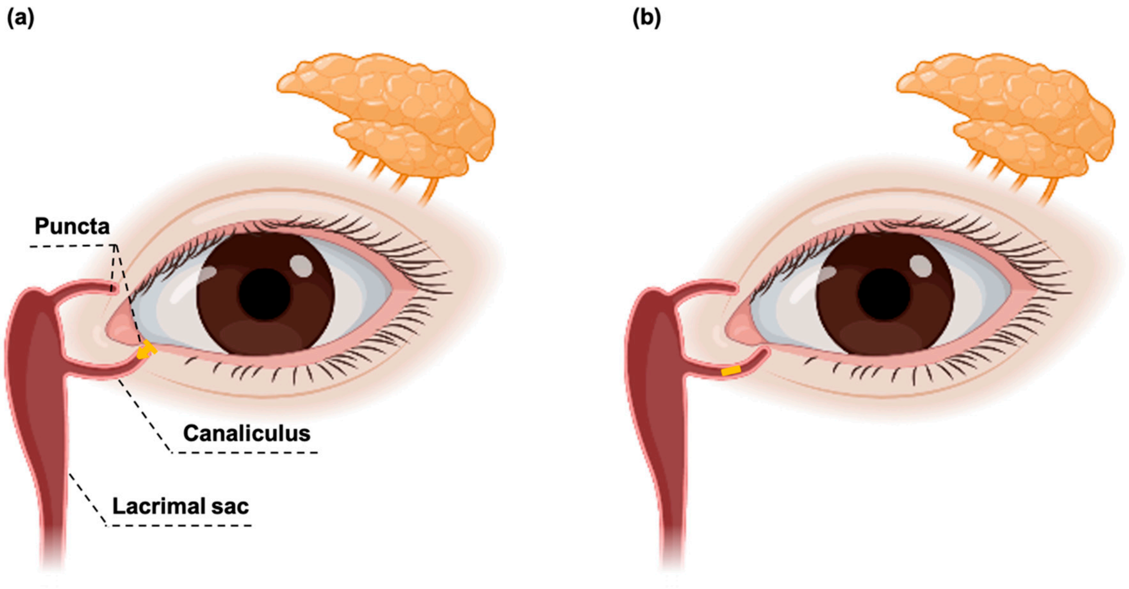

Figure S1. Schematic illustration of punctal plug (**a**) in the punctum and (**b**) in the canaliculus of the eye.
